# Effects of Postoperative Gum Chewing on Recovery of Gastrointestinal Function Following Laparoscopic Gynecologic Surgery: Systematic Review and Meta-Analysis of Prospective Studies

**DOI:** 10.3390/jcm13102851

**Published:** 2024-05-12

**Authors:** Thunwipa Tuscharoenporn, Kittithat Uruwankul, Kittipat Charoenkwan

**Affiliations:** 1 Department of Obstetrics and Gynecology, Faculty of Medicine, Chiang Mai University, Chiang Mai 50200, Thailand; thunwipatus@gmail.com; 2 Faculty of Medicine, Chiang Mai University, Chiang Mai 50200, Thailand; kittithat_uruwankul@cmu.ac.th

**Keywords:** gum chewing, gynecology, laparoscopy

## Abstract

**Background:** Chewing gum, considered a form of sham feeding, has been shown to improve intestinal motor and secretory function in various types of abdominal surgery. We conducted this systematic review to evaluate the effects of postoperative gum chewing on the recovery of gastrointestinal function after laparoscopic gynecologic surgery. **Methods:** We performed a comprehensive literature review of all prospective clinical trials in PubMed, Embase, and a reference list of relevant studies from the inception to 11 March 2024, comparing postoperative gum chewing versus no gum chewing following laparoscopic gynecologic surgery regardless of indications and setting without language restriction. The primary outcome was the time to the presence of bowel sounds and the time to the first passage of flatus. Cochrane’s risk of bias tool was used to assess the risk of bias in included studies. **Results:** Nine studies with a total of 1011 patients were included. Overall, three studies were categorized as having a low risk of bias, three had some concerns, and three exhibited a high risk of bias. The time to the presence of bowel sounds (mean difference [MD] −2.66 h, 95% confidence interval [CI] −3.68 to −1.64, *p* < 0.00001) and time to the first passage of flatus (MD −4.20 h, 95% CI −5.79 to −2.61, *p* < 0.00001) was significantly shorter in the gum-chewing group. There was no statistical difference between the two groups with regard to the time to the first defecation (MD −6.52 h, 95% CI −15.70 to 2.66, *p* = 0.16), time to the first postoperative mobilization (MD 24.05 min, 95% CI −38.16 to 86.26, *p* = 0.45), postoperative ileus (MD 0.68, 95% CI 0.39 to 1.19, *p* = 0.17), and length of hospital stay (MD −0.05 day, 95% CI −0.14 to 0.04, *p* = 0.28). **Conclusions:** Gum chewing following laparoscopic gynecologic surgery appears to promote the recovery of gastrointestinal function, as evidenced by a reduced time to the presence of bowel sounds and the first passage of flatus.

## 1. Introduction

Abdominal surgery is a primary treatment for both benign and malignant gynecologic conditions. Following abdominal surgery, it is typical for a certain level of paralytic ileus to occur. This can be attributed to various factors such as local and generalized intestinal inflammation as well as the release of inhibitory neurotransmitters from the bowel wall due to bowel manipulation, the use of perioperative opioids, and the stimulation of afferent reflexes associated with peritoneal irritation [1,2,3]. This frequently results in nausea, vomiting, abdominal distention, delayed bowel movement, inability to eat, prolonged hospital stay, and increased cost [3].

Laparoscopic surgery, characterized by less physical manipulation, smaller incisions, and a lesser inflammatory response compared to laparotomy, facilitates quicker bowel function recovery and reduces the incidence of ileus [4]. Additionally, the reduced pain and smaller incisions associated with laparoscopy enhance patient mobility, further decreasing the risk of ileus. However, laparoscopy involves insufflating the abdominal cavity with CO_2_, which increases intra-abdominal pressure. This can temporarily impair intestinal blood flow and motility, potentially inducing ileus. Moreover, CO_2_ insufflation may cause acidosis and electrolyte imbalances, adversely affecting intestinal smooth muscle function and delaying the recovery of normal gut function [5].

The Enhanced Recovery After Surgery (ERAS) protocol is designed to minimize the physiological stress of surgery, enhance the recovery process, and facilitate earlier hospital discharge [6]. Key components of the ERAS protocol include preoperative counseling, optimized fluid management, minimized fasting, early mobilization, and early enteral nutrition. Early enteral feeding plays a crucial role by stimulating gut motility and maintaining the integrity of the gut mucosa. It is essential for providing adequate nutrition, which is vital for healing and recovery [7]. Furthermore, early feeding can reduce the incidence of infections by maintaining gut barrier function and reducing bacterial translocation. However, challenges remain as some patients may experience nausea, vomiting, or bloating, especially when gastrointestinal function is slow to recover. Additionally, there is a risk of aspiration in patients with reduced consciousness or those who are unable to protect their airway [8].

Chewing gum, considered a form of sham feeding, has been suggested to improve intestinal motor and secretory function. This is because chewing activates the cephalic-vagal reflex, which in turn stimulates intestinal myoelectric activity and triggers the release of various substances such as gastrin, neurotensin, pancreatic polypeptides, and duodenal enzymes [9,10]. The safety and benefits of postoperative gum chewing have been demonstrated following various types of abdominal surgery, including cesarean section [11,12,13,14], colectomy [15], and radical cystectomy [16]. Importantly, previous research has explored the effects of gum chewing on intestinal function after laparoscopic gynecologic surgery. A recent meta-analysis by Douligeris et al. demonstrated the positive effects of postoperative gum chewing on gastrointestinal function following laparoscopic gynecological surgery. Building upon this foundation, we aim to expand the evidence base further by including additional prospective studies and conducting pooled meta-analyses stratified by risk of bias subgroups. This approach would provide a comprehensive understanding of the effects of gum chewing in this specific surgical context [17].

This systematic review evaluated the evidence supporting the use of postoperative gum chewing to improve recovery from intestinal ileus after gynecologic laparoscopic surgery, regardless of the setting (elective or emergency) or underlying conditions (benign or malignant).

## 2. Materials and Methods

This systematic review protocol was registered with PROSPERO (CRD42017058754). This review was conducted and reported according to the PRISMA statement [18].

### 2.1. Criteria for Considering Studies for This Review

The criteria for study inclusion in this review are as follows:

1. Population: Women undergoing gynecologic laparoscopic surgery, regardless of setting (elective or emergency) and underlying conditions (benign or malignant).
2. Intervention: Postoperative gum chewing in addition to standard postoperative care.
3. Comparator: Standard postoperative care.
4. Study design: Prospective studies published as full-text articles in any language and geographic location. There was no limit on the number of study participants or the time of publication.

### 2.2. Types of Outcome Measures

The primary outcomes were the time to the presence of bowel sounds and the time to the first passage of flatus. Secondary outcomes included the time to the first defecation, the time to the first postoperative mobilization, the occurrence of postoperative ileus (defined as mild-to-severe intestinal obstruction, abdominal distension, nausea, and vomiting), and the length of hospital stay.

### 2.3. Search Methods for Identification of Studies

A systematic literature search was conducted through PubMed and Embase using search concepts: gum chewing and surgery, with relevant search terms to form search strategies specific to each database. The detailed search strategies were as follows:

- PubMed was searched from inception to 11 March 2024. The PubMed search strategy was as follows:
  “surger*” [Text Word] OR “laparos*” [Text Word] OR “surgical procedures, operative” [MeSH Terms] (4,862,000)
  “Chewing Gum” [Text Word] OR “gum chewing” [Text Word] OR “Chewing Gum” [MeSH Terms] (3491)
  1. AND 2. (372)
- Embase was searched from inception to March 11, 2024. The EMBASE search strategy was as follows:
  surg* OR laparos* OR ‘laparoscopy’/exp OR ‘laparoscopy’ OR ‘laparoscopic surgery’/exp OR ‘laparoscopic surgery’ (7,144,372)
  ‘gum chewing’ OR ‘chewing gum’ (4212)
  1. AND 2. (696)

In addition, ClinicalTrials.gov was searched for ongoing unpublished trials. We also searched the citation lists of included studies, relevant publications, systematic reviews, and review articles.

### 2.4. Study Selection and Data Extraction

Studies were considered eligible if they met the inclusion criteria. The titles and abstracts of articles found in the search were screened by two authors (TT and KU), who discarded studies that were clearly ineligible. Then, the full-text articles of the eligible studies were obtained and were independently assessed by the three authors for inclusion. Any disagreements were resolved by discussion. Subsequently, the authors independently extracted information using the pre-designed data extraction form, with discrepancies resolved by discussion. Further information was sought from the authors of the original studies where articles contained insufficient data to make a decision about eligibility, methodological quality, and outcomes of interest.

### 2.5. Risk of Bias Assessment

The two authors (TT and KC) independently examined the risk of bias in the included studies based on the assessment tool recommended by Cochrane Collaboration [19]. The tool is a critical evaluation based on six different domains, including sequence generation, allocation concealment, blinding, incomplete outcome data, selective outcome reporting, and other sources of bias. Discrepancies were resolved by discussion.

### 2.6. Statistical Analysis

Statistical analysis was performed by using the Cochrane Review Manager (RevMan) software [20]. Heterogeneity between the outcomes of the included studies was examined by inspecting the forest plot, performing formal statistical tests of homogeneity of 2 × 2 tables (Chi^2^ test), and calculating the I^2^ statistic. A *p*-value of the heterogeneity Chi^2^ test of <0.1 and an I^2^ value of >50% indicated significant heterogeneity. For the meta-analysis of continuous outcomes, e.g., the time to the presence of bowel sounds, time to the first passage of flatus, time to the first defecation, time to the first postoperative mobilization, and length of hospital stay, the weighted mean difference (MD) with a 95% confidence interval (CI) was estimated. For the studies in which continuous outcomes were expressed as the median with range, the data were converted to the mean with standard deviation (SD) by employing the procedure proposed by Luo et al. [21] and Wan et al. [22]. For a dichotomous outcome (postoperative ileus), the results from each study and the pooled analysis were expressed as relative risks with a 95% CI. For the meta-analysis of each outcome, if significant heterogeneity existed, a random-effects model was used; otherwise, the fixed-effects model was employed. Sensitivity analyses were performed to explore whether the effects of gum chewing on the recovery of bowel function varied by the risk of bias status of the included studies.

## 3. Results

### 3.1. Characteristics of Included Studies

**Figure 1:**
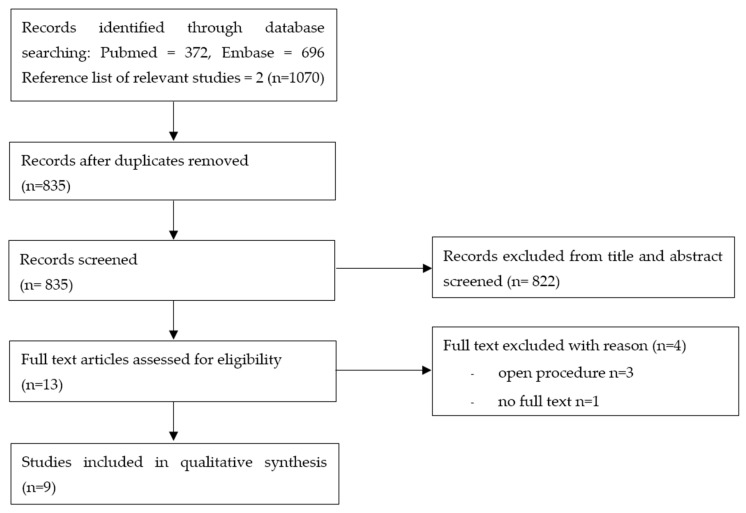
PRISMA flow diagram.

The systematic literature search yielded 835 studies after de-duplication. Thirteen studies met the inclusion criteria. Three studies examined women who underwent open procedures, not laparoscopy, and we could not obtain the full text of one study. Therefore, these four studies were excluded. Nine studies involving 1011 patients in total were included in this analysis (Table 1). Of these, 503 patients were in the gum-chewing group, and 508 were in the control group. Figure 1 presents the PRISMA flow diagram.

The included studies were distributed across the following countries: China (four studies), Turkey (two studies), Austria (one study), India (one study), and Indonesia (one study). The sample size varied from 60 to 234. The majority of the studies involved participants with benign diseases except Gong et al. [24], in which participants with malignant conditions were also included. There were limited data on preoperative bowel preparation. The gum chewing regimens, although similar, varied in detail across studies in terms of postoperative starting time, frequency, duration, and discontinuation time. The most common schedule, however, was chewing gum every two hours for 15 min (six studies) until flatus (seven studies). The time to the initial passage of flatus constituted a primary outcome measure across all studies incorporated in the review, whereas the time until the manifestation of audible bowel sounds represented a primary outcome in six of the included studies. Table 2 displays the characteristics of participants in the included studies.

### 3.2. The Risk of Bias in the Included Studies

**Figure 2:**
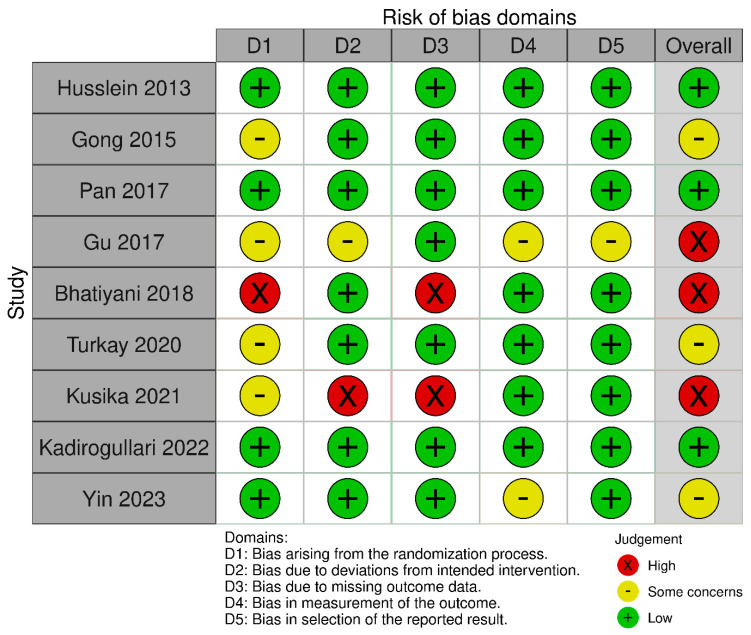
Traffic light plot [23,24,25,26,27,28,29,30,31].

**Figure 3:**
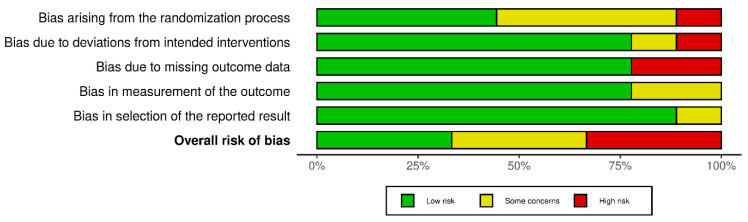
Summary plot.

Figure 2 displays the risk of bias for each included study, classified by bias domains, and Figure 3 presents a cumulative assessment of the potential biases encountered. The bias in the measurement of outcomes and selection of reported results was considered to be low. However, biases or some concerns arising from the randomization process, deviations from intended interventions, and missing outcome data contributed to the high overall risk of bias in some studies. Overall, three studies were categorized as having a low risk of bias [23,25,30], three had some concerns [24,28,31], and three exhibited a high risk of bias [26,27,29].

### 3.3. Effects of Interventions

**Figure 4:**
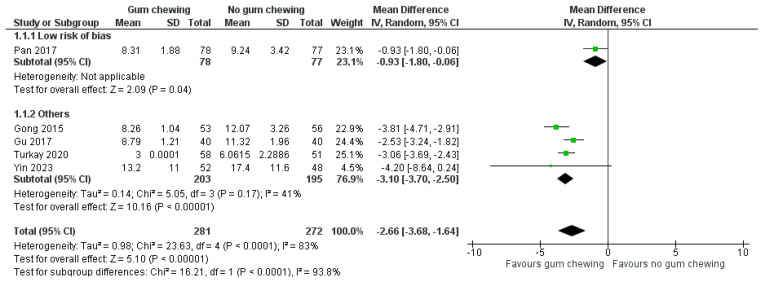
Time to the presence of bowel sound (hours) [24,25,26,28,31].

Time to the presence of bowel sounds (Figure 4)

Five studies involving 553 participants reported the time to the presence of bowel sounds. One study had a low risk of bias [25], while others had more than a low risk of bias [24,26,28,31]. Overall, participants in the gum-chewing group experienced a significantly shorter time to the presence of bowel sounds compared to the control group (MD −2.66 h, 95% CI −3.68 to −1.64, *p* < 0.00001).

**Figure A1:**
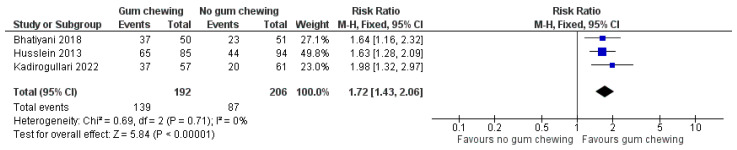
Presence of bowel sounds 3 h after surgery [23,27,30].

**Figure A2:**
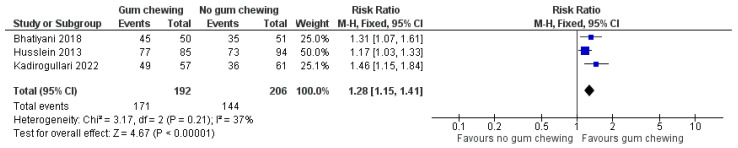
Presence of bowel sounds 5 h after surgery [23,27,30].

**Figure A3:**
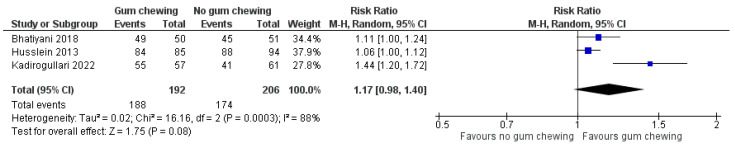
Presence of bowel sounds 7 h after surgery [23,27,30].

Three studies involving 398 participants reported the proportion of participants with audible bowel sounds at 3, 5, and 7 h postoperation. There was a significantly higher proportion of patients with audible bowel sounds at 3 and 5 h in the gum-chewing group (RR 1.72, 95% CI 1.43 to 2.06, *p* < 0.00001, and RR 1.28, 95% CI 1.15 to 1.41, *p* < 0.00001, respectively). However, at 7 h postoperation, the higher rate of the audible bowel sounds in the gum-chewing group was not statistically significant (RR 1.17, 95% CI 0.98 to 1.40, *p* = 0.08). The presence of bowel sounds at 3 h, 5 h, and 7 h postoperation is displayed in Figure A1, Figure A2 and Figure A3.

**Figure 5:**
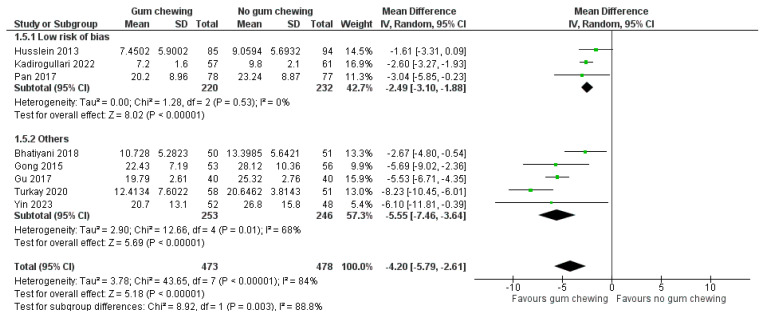
The time to the first passage of flatus (hours) [23,24,25,26,27,28,30,31].

Time to the first passage of flatus (Figure 5)

Eight studies involving 951 participants provided data for this outcome that could be pooled in a meta-analysis. Three studies were categorized as a low risk of bias [23,25,30], three had some concerns [24,28,31], and two were deemed high-risk [26,27]. Overall, gum chewing significantly reduced the time to the first passage of flatus compared to the control group (MD −4.20 h, 95% CI −5.79 to −2.61, *p* < 0.00001). This effect remained statistically significant but less pronounced when only studies with a low risk of bias were considered (MD −2.49 h, 95% CI −3.10 to −1.88, *p* < 0.00001). In Kusika et al.’s study, in which this outcome was reported as the mean with range, flatus occurred significantly sooner in the gum-chewing group (15.95 h vs. 45.05 h, *p* < 0.001) [29].

**Figure 6:**
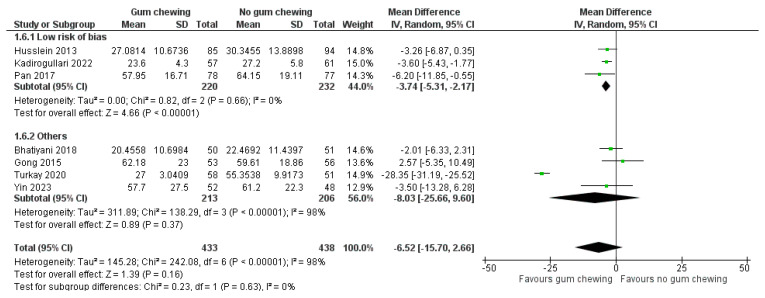
The time to the first defecation (hours) [23,24,25,27,28,30,31].

Time to the first defecation (Figure 6)

Seven studies involving 871 participants provided data for a meta-analysis of this outcome. Among these, three studies were categorized as having a low risk of bias [23,25,30], while the remaining four studies were classified as having some concern or a high risk of bias [24,27,28,31]. Overall, a statistically significant difference in the time to the first defecation between the groups could not be demonstrated (MD −6.52 h, 95% CI −15.70 to 2.66, *p* = 0.16). Of note, when only studies with a low risk of bias were considered, the gum-chewing group had a significantly shorter time to the first defecation (MD −3.74 h, 95% CI −5.31 to −2.17, *p* < 0.00001).

**Figure 7:**
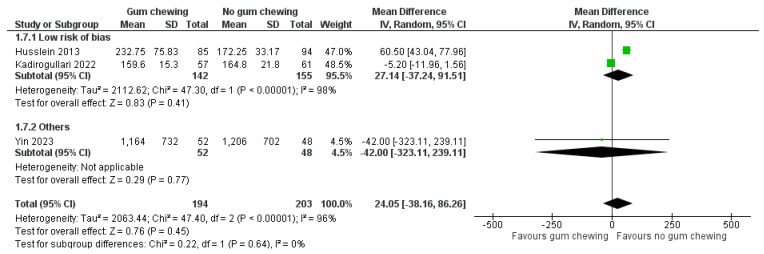
The time to the first postoperative mobilization (minutes) [23,30,31].

Time to the first postoperative mobilization (Figure 7)

Three studies involving 397 participants provided data for a pooled analysis of this outcome. Two studies were classified as a low risk of bias [23,30], and one study had some concerns regarding bias [31]. Overall, there was no significant difference in the time to the first postoperative mobilization between the gum-chewing group and the control group (MD 24.05 min, 95% CI −38.16 to 86.26, *p* = 0.45).

**Figure 8:**
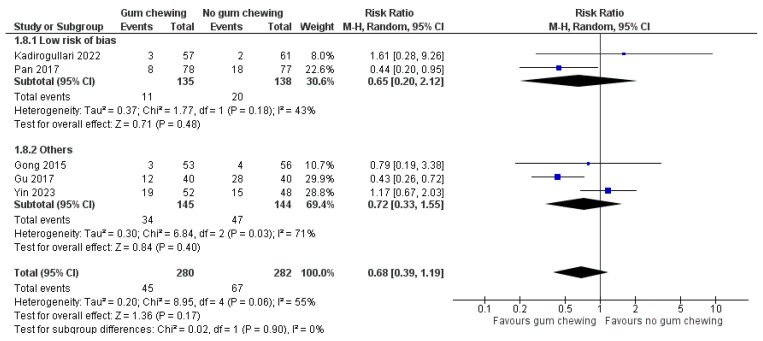
Postoperative ileus [24,25,26,30,31].

Postoperative ileus (Figure 8)

Data from five studies involving 562 participants were pooled in a meta-analysis of this outcome. While two studies had a low risk of bias [25,30], two had some concerns [24,31], and one was considered high-risk [26]. The analysis showed no significant difference in the number of patients experiencing postoperative ileus between the gum-chewing and control groups (RR 0.68, 95% CI 0.39 to 1.19, *p* = 0.17).

**Figure 9:**
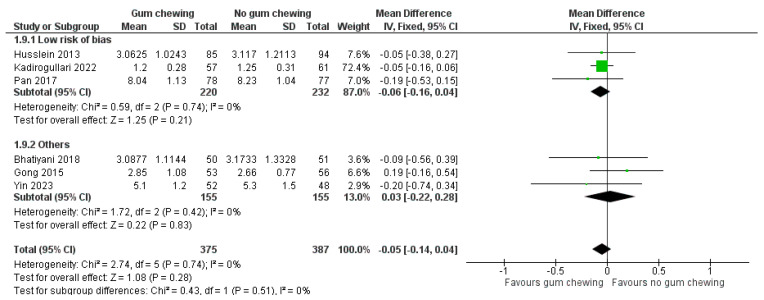
Length of hospital stay (days) [23,24,25,27,30,31].

Length of hospital stay (Figure 9)

Six studies involving 762 participants provided data for a meta-analysis of this outcome. Three studies were classified as having a low risk of bias [23,25,30], two had some concerns [24,31], and one was considered as having a high risk of bias [27]. There was no significant difference in the length of hospital stay between the gum-chewing and the control groups (MD −0.05 day, 95% CI −0.14 to 0.04, *p* = 0.28). In the study by Kusika et al., the duration of hospital stay was reported using the mean and range. It was found that patients in the gum-chewing group experienced a significantly shorter hospital stay compared to the control group (15.50 h vs. 45.50 h (*p* < 0.001) [29].

## 4. Discussion

### 4.1. Main Findings

This systematic review and meta-analysis of nine studies suggest that gum chewing significantly accelerates the postoperative recovery of gastrointestinal function in women undergoing laparoscopic gynecologic surgery. The evidence demonstrates a shorter time to the presence of bowel sounds and an earlier passage of the first flatus in the gum-chewing group compared to the control group. However, the meta-analysis did not reveal any significant benefits of gum chewing regarding the time to the first postoperative mobilization, the occurrence of postoperative ileus, or the length of hospital stay. When considering only studies with a low risk of bias in the sensitivity analysis, the beneficial effects of gum chewing on the return of bowel sounds and passage of flatus, although less pronounced, remained statistically significant. Moreover, the analysis revealed that gum chewing expedited the time to the first defecation in the intervention group.

### 4.2. The Strengths and Limitations of the Study

This review has recognizable strengths. First, a systematic approach to searching multiple databases, including both the published and unpublished literature, was adopted to ensure the identification of all relevant studies without language restriction. A study originally published in Chinese was translated and included [26]. Second, well-defined inclusion and exclusion criteria for selecting studies were employed. Third, a thorough evaluation of the methodological quality and potential sources of bias in the included studies, using the established Cochrane risk of bias tool, was carefully performed. Fourth, the ability to pool adequate data from the included studies and perform meta-analyses on primary outcomes of interest increases the statistical power and precision of the effect estimates. Additionally, sensitivity analyses that exclude studies with a high risk of bias further strengthened the robustness of the findings.

However, some limitations exist that warrant consideration. As depicted in Figure 2 and Figure 3, the overall risk of bias varied across the included studies. The risk of bias observed was primarily due to the randomization process, deviations from the intended interventions, and missing outcome data. Several studies lacked detailed information on the randomization method, while others did not provide sufficient details on the blinding of participants and personnel involved in delivering the intervention. Furthermore, some studies did not adequately address missing data or provide clear information on the handling of incomplete outcomes. To address this limitation, we performed sensitivity analyses by categorizing the included studies based on their overall risk of bias, allowing for a more nuanced interpretation of the findings. Additionally, significant heterogeneity was observed among the included studies concerning factors such as preoperative bowel preparation protocols, intraoperative anesthesia techniques, postoperative diet timing and composition, and the gum-chewing schedule. Also, it should be noted that while including both benign and malignant gynecologic conditions in the study population increases the generalizability of the findings to a wider range of clinical scenarios, it could introduce the risk of bias and complicate the interpretation of the findings. However, in the only study that included laparoscopic surgery performed for malignant indications, only 11 out of 109 participants (approximately 10%) had major procedures usually performed for malignant conditions [24]. As the great majority of the included participants underwent laparoscopic surgery for benign gynecologic conditions, the findings of this review may not be applicable to those with malignant diseases.

### 4.3. Interpretation in Light of Other Evidence

Some surgeons have expressed concerns that early enteral feeding following gynecologic surgery could lead to severe complications such as paralytic ileus, vomiting, aspiration pneumonia, and wound complications. However, a Cochrane systematic review has shown that early feeding initiation following major abdominal gynecologic surgery, whether for benign or malignant conditions, seemed to pose no additional risk of gastrointestinal complications or other postoperative issues. This strategy offers advantages such as a quicker restoration of bowel function, a reduced incidence of infectious complications, shorter hospitalization, and increased patient satisfaction [7]. Nonetheless, some patients may not feel ready to start eating within the first few days after surgery. This is where gum chewing may come in.

A review that included several randomized controlled trials evaluated the effects of chewing gum on the postoperative recovery of gastrointestinal function after different types of surgeries. The review’s findings suggested that chewing gum can effectively accelerate the postoperative recovery of gastrointestinal function, particularly in non-gastrointestinal surgeries like gynecologic surgery, cesarean section, hepatic surgery, vascular surgery, and urologic surgery. However, there is still controversy regarding the benefits of gum chewing after gastrointestinal surgery [32]. Another Cochrane systematic review has demonstrated the potential advantages of utilizing postoperative chewing gum to enhance the recuperation of gastrointestinal function. Nonetheless, the studies included in that review have mainly concentrated on colorectal surgery and cesarean section and predominantly comprised small-scale, low-quality trials with methodological constraints. It is also crucial to acknowledge that numerous elements of the Enhanced Recovery After Surgery (ERAS) initiative also address postoperative ileus, potentially diminishing the supplementary benefits of gum chewing when integrated with ERAS protocols [33]. In another meta-analysis examining the effects of gum chewing on gastrointestinal recovery following gynecologic surgery, eight randomized controlled trials conducted from 2013 to 2017 involving a total of 1077 women were included. Gum chewing significantly reduced the time to the first flatus and defecation after gynecologic surgery and the length of hospital stay [34]. Recently, a systematic review including six randomized controlled trials with 669 participants assessed the impact of gum chewing on postoperative ileus following gynecologic cancer surgery. Chewing gum after surgery for gynecological cancer helps promote the faster recovery of gastrointestinal function, reducing the chances of postoperative ileus. Gum chewing is also associated with a quicker occurrence of the first flatus and bowel movement, as well as shorter hospital stays without complications [35]. While the systematic reviews suggest potential benefits of gum chewing in promoting postoperative gastrointestinal recovery, one could doubt the impact of this intervention, specifically on laparoscopic gynecologic surgery due to the minimally invasive nature of these procedures, which may result in less disruption of gastrointestinal function compared to more invasive surgeries. In a recent meta-analysis on the effect of postoperative gum chewing on gastrointestinal function after laparoscopic gynecological surgery, including 5 studies with 670 participants, gum chewing significantly reduced the time to the first bowel sound and the time to the first passage of flatus. However, there was no significant difference between the two study groups with regard to the time to the first defecation, the time to the first postoperative mobilization, the length of hospital stay, and the risk of postoperative bowel obstruction [17].

The role of specific ingredients in chewing gum, particularly sorbitol, warrants attention. Sorbitol, commonly used as a sweetener in gum, has osmotic properties that can stimulate bowel movements and lead to diarrhea if overconsumed. It was hypothesized that hexitols, like sorbitol in chewing gum, might reduce postoperative ileus by increasing intestinal motility [36]. Additionally, a systematic review highlighted the gastrointestinal effects of polyols, including their potential to cause osmotic diarrhea, relevant for post-surgery bowel function [37]. Moreover, a study and case report highlighted sorbitol’s role in inducing osmotic diarrhea and explored preventive strategies, underscoring the ingredient’s dose-dependent impact [38,39]. It is essential for healthcare providers to carefully consider and properly inform the patient regarding this relevant issue when implementing the gum chewing protocol.

### 4.4. Implications of the Review Findings

Our study findings suggest that gum chewing can potentially facilitate the recovery of gastrointestinal function following laparoscopic gynecologic surgery without any reported complications. Gum chewing emerges as an affordable, readily accessible, and well-tolerated postoperative intervention. However, the high heterogeneity observed among the included studies indicates potential variability in patient responses to gum chewing, highlighting the need for further research to understand the factors influencing its effectiveness. Also, future studies could focus on standardizing the gum-chewing protocol post-gynecological surgery to determine the optimal frequency and duration for maximum benefits.

## 5. Conclusions

Gum chewing after laparoscopic gynecologic surgery appears to promote the recovery of gastrointestinal function, as evidenced by the reduced time to the presence of bowel sounds and the first passage of flatus.

This evidence supports the development or revision of postoperative care protocols for laparoscopic gynecologic surgeries. Healthcare providers, including nurses and patient educators, can utilize this information to inform patients about the benefits of gum chewing post-surgery. Such findings encourage collaboration among healthcare professionals involved in patient care, potentially reducing hospital stays and associated costs.

## Figures and Tables

**Table 1:** Characteristics of included prospective studies.

Study, Year, Country	Type of Studies	Participants Number (Gum Chewing/ Control)	Type of Surgery	Preoperative Bowel Preparation	Gum Chewing Schedule	Outcomes
Husslein et al., 2013 [23], Austria	RCT	179 (85/94)	Gynecologic laparoscopic surgery for benign conditions under general anesthesia	No bowel preparation	Started chewing a commercially available sugarless gum 2 h after surgery, every 2 h for 15 min. Stopped after flatus.	Primary: TBS, TFF Secondary outcome: TFD, TPM, LOS, patient satisfaction, potential side effects of postoperative gum chewing, and the potential effect of gum chewing on postoperative pain therapy
Gong et al., 2015 [24], China	RCT	109 (53/56)	Elective gynecologic laparoscopic surgery for benign and malignant disease	Oral sodium phosphate enema catharsis and a preoperative mechanical anal wash	Started chewing mint-flavored, xylitol-containing sugarless gum 6 h after surgery, 3 times a day for 30 min at each session. Stopped after flatus.	Primary outcome: TFF Secondary outcome: TBS, TFD, LOS, POI (mild obstruction = anorexia, nonpersistent nausea or vomiting; severe obstruction = abdominal distension, >4 times within 24 h of vomiting, cannot tolerate oral intake, and the need for decompression)
Pan et al., 2017 [25], China	RCT	234 (Gum chewing and semi-liquid diet 78 /Semi-liquid-only diet 77/ Liquid diet 79	Elective gynecologic laparoscopic surgery	No data available	Started chewing a commercially available xylitol-containing sugar-free gum when fully awake, every 2 h (except from 20.00 to 8.00) for 15 min. Stopped after flatus.	Primary outcome: TBS, time to first regular postoperative bowel sounds, TFF, TFD Secondary outcome: LOS, the incidence of hunger, nausea, vomiting, abdominal distension, and intestinal obstruction, preoperative serum level of gastrin both 12 and 24 h after surgery
Gu et al., 2017 [26], China	RCT	80 (40/40)	Elective gynecologic laparoscopic surgery	No data available	Started chewing gum, based on patient’s candy brand and taste preferences, after surgery 3 times a day for 30 min.	TBS, TFF, nausea, vomiting, abdominal distension, complication rate%, time to tolerating liquid diet
Bhatiyani et al., 2018 [27], India	Prospective studies	101 (50/51)	Gynecologic laparoscopic surgery for benign conditions under general anesthesia	No data available	Started chewing gum 2 h after surgery every 2 h for 15 min. Stopped after flatus.	Primary outcome: TBS, TFF Secondary outcome: TFD, TPM, patient satisfaction, and potential side effects of gum chewing
Turkay et al., 2020 [28], Turkey	RCT	109 (58/51)	Total laparoscopic hysterectomy for benign gynecologic conditions	Bowel preparation with 28.5 g NaH2P + 10.5 g Na2HP	Started chewing sugar-free peppermint-flavored gum 2 h after surgery every 2 h for 15 min. Stopped after flatus.	Primary outcome: TBS, TFF, TFD Secondary outcome: The amount of postoperative analgesics needed and the pain-related VAS score at 6, 12, and 24 h, postoperatively
Kusika et al., 2021 [29], Indonesia	Prospective studies	60 (30/30)	Gynecologic laparoscopic surgery	No data available	Started chewing sugar-free gum right after surgery 5 times with 2 h intervals for 15 min	TFF, TBS, LOS
Kadirogullari et al., 2022 [30], Turkey	RCT	118 (57/61)	Elective total laparoscopic hysterectomy for benign gynecologic conditions	No data available	Started chewing sugar-free gum 4 h after surgery every 2 h for 15 min. Stopped after flatus.	Primary outcome: TFF Secondary outcome: TBS, TFD, TPM, LOS, POI (mild intestinal obstruction = anorexia, non-repeating nausea, and vomiting; severe intestinal obstruction = flatulence for 24 h, vomiting repeatedly more than 4 times, the intolerance of oral feeding, and the need for decompression)
Yin et al., 2023 [31], China	RCT	100 (52/48)	Elective gynecologic single port laparoscopic surgery for benign conditions	Oral sodium phosphate	Started chewing commercial sugar-free gum (contain xylitol) 2 h after surgery every 6 h for 15 min. Stopped after flatus.	Primary outcome: TFF Secondary outcome: TBS, TFD, LOS, TPM, postop NRS score at 6, 12, and 24 h, POI, postoperative 6 h nausea and abdominal distension

Abbreviations: TBS = time to the presence of bowel sounds, TFF = time to the first passage of flatus, TFD = time to the first defecation, TPM = time to the first postoperative mobilization, POI = postoperative ileus, LOS = length of hospital stay.

**Table 2:** The characteristics of participants in the included studies (gum chewing vs. control).

Study, Year, Country	Age (Years)	BMI (kg/m^2^)	Duration of Surgery (Mins)	Type of Laparoscopic Surgery (%)
Husslein et al., 2013 [23], Austria	40 (21–75) vs. 42 (19–74)	23.1 (17.8–36.6) vs. 23.6 (16.1–38.2)	43 (10–149) vs. 47 (10–162)	Unilateral SO or salpingectomy: 13 (15) vs. 12 (13) BSO: 3 (4) vs. 9 (10) TLH with or without BSO: 10 (12) vs. 9 (10) Myomectomy: 7 (8) vs. 7 (7) Ovarian cystectomy: 24 (28) vs. 22 (23) Diagnostic laparoscopy with or without removal of endometriosis: 28 (33) vs. 35 (37)
Gong et al., 2015 [24], China	39.55 ± 10.25 vs. 39.41 ± 10.64	No data available	83.4 ± 45.6 vs. 67.2 ± 34.8	Minor surgery (laparoscopy, accessories surgery, uterine fibroids excavation technique, EMS fulguration): 39 (74) vs. 40 (71) Moderate surgery (hysterectomy surgery): 8 (15) vs. 11 (20) Major surgery (hysterectomy fascia resection, hysterectomy wide excision, uterine wide excision): 6 (11) vs. 5 (9)
Pan et al., 2017 [25], China	42.76 ± 7.41 vs. 43.94 ± 7.11	No data available	72.6 ± 29.4 vs. 79.2 ± 33.6	Ovarian cystectomy: 22 (28) vs. 19 (25) Adnexectomy: 5 (6) vs. 7 (9) Myomectomy: 22 (28) vs. 23 (30) Subtotal hysterectomy: 9 (12) vs. 10 (13) Hysterectomy: 20 (26) vs. 18 (23)
Gu et at., 2017 [26], China	Overall cases 34.22 ± 2.33 (21–56)	No data available	No data available	Overall cases Ovarian cyst resection: 43 (54) cases Tubal incision or resection: 37 (46) cases
Bhatiyani et al., 2018 [27], India	30 (24–56) vs. 32 (21–58)	23.1 (17.8–36.6) vs. 23.6 (16.1–38.2)	90 (30–200) vs. 120 (30–228)	TLH: 6 (12) vs. 4 (8) MTP with TL: 20 (40) vs. 18 (35) Unilateral SO or salpingectomy: 7 (14) vs. 9 (18) Diagnostic Hysterolaparoscopy: 17 (34) vs. 19 (37)
Turkay et al., 2020 [28], Turkey	48 (40, 52) vs. 48 (46, 55) *MD (Q1, Q3)	26.59 (23.53, 30.44) vs. 27.80 (26.00, 30.40) *MD (Q1, Q3)	86.88 ± 14.16 vs. 84.96 ± 19.35	TLH + BSO: 35 (60) vs. 28 (55) TLH + BS: 20 (35) vs. 21 (41) TLH + USO: 1 (2) vs. 1 (2) TLH: 2 (3) vs. 1 (2)
Kusika et al., 2021 [29], Indonesia	23–44 vs. 21–42	No data available	150 vs. 150	Laparoscopy and hysteroscopy Laparoscopic cystectomy Hysteroscopic resection of polyp
Kadirogullari et al., 2022 [30], Turkey	48.3 ± 8.6 vs. 46.7 ± 9.2	24.6 ± 5.3 vs. 25.1 ± 6.3	83.3 ± 12.8 vs. 78.4 ± 15.6	TLH benign conditions
Yin et al., 2023 [31], China	42.8 ± 8.0 vs. 44.6 ± 6.4	23.4 ± 2.9 vs. 22.3 ± 4.3	132 ± 60 vs. 138 ± 48	Myomectomy: 33 (64) vs. 27 (56) TLH + BS: 10 (19) vs. 14 (29) TLH + BSO: 8 (15) vs. 5 (10) Ovarian cystectomy: 1 (2) vs. 2 (4)
