# Correction: Tuscharoenporn et al. Effects of Postoperative Gum Chewing on Recovery of Gastrointestinal Function Following Laparoscopic Gynecologic Surgery: Systematic Review and Meta-Analysis of Prospective Studies. *J. Clin. Med.* 2024, *13*, 2851

**DOI:** 10.3390/jcm14072252

**Published:** 2025-03-26

**Authors:** Thunwipa Tuscharoenporn, Kittithat Uruwankul, Kittipat Charoenkwan

**Affiliations:** 1 Department of Obstetrics and Gynecology, Faculty of Medicine, Chiang Mai University, Chiang Mai 50200, Thailand; thunwipatus@gmail.com; 2 Faculty of Medicine, Chiang Mai University, Chiang Mai 50200, Thailand; kittithat_uruwankul@cmu.ac.th

**Text Correction**

1. There was an error in the original publication [1]. There is a probable misclassification of the included study types identified in the review, specifically concerning the studies by Bhatiyani et al. (2018) and Kusika et al. (2021), which were described as randomized controlled trials (RCTs). They should have been classified adequately as prospective studies. A correction has been made to the Title, Abstract (Methods and Results sub-sections), Methods (Criteria for Considering Studies for This Review sub-section), Results (Characteristics of Included Studies sub-section), and Discussion (Main Findings sub-section) accordingly. In addition, the column “Type of studies” has been added to Table 1 to differentiate the types of the included studies.
2. To properly contextualize this review’s findings within the existing body of literature, statements about prior research have been added to the Introduction section on Page 2 and the Discussion section on pages 13 and 14.

**The Statement Added to the Introduction Section (Page 2)**

Importantly, previous research has explored the effects of gum chewing on intestinal function after laparoscopic gynecologic surgery. A recent meta-analysis by Douligeris et al. demonstrated the positive effects of postoperative gum chewing on gastrointestinal function following laparoscopic gynecological surgery. Building upon this foundation, we aimed to expand the evidence base further by including additional prospective studies and conducting pooled meta-analyses stratified by risk of bias subgroups. This approach was expected to provide a comprehensive understanding of the effects of gum chewing in this specific surgical context.

**The Statement Added to the Discussion Section (Pages 13–14)**

In a recent meta-analysis on the effect of postoperative gum chewing on gastrointestinal function after laparoscopic gynecological surgery, including five studies with 670 participants, gum chewing significantly reduced the time to the first bowel sound and the time to the first passage of flatus. However, there was no significant difference between the two study groups with regard to the time to the first defecation, the time to the first postoperative mobilization, the length of hospital stay, and the risk of postoperative bowel obstruction [17].

**Figure/Table Legend**

In the original publication, there was a mistake in the legend for Table 1. Characteristics of included randomized controlled trials. The correct legend appears below. The correct legend: “Characteristics of included prospective studies”.

The authors state that the scientific conclusions are unaffected. This correction was approved by the Academic Editor. The original publication has also been updated.
